# CIViC is a community knowledgebase for expert crowdsourcing the clinical interpretation of variants in cancer

**DOI:** 10.1038/ng.3774

**Published:** 2017-01-31

**Authors:** Malachi Griffith, Nicholas C Spies, Kilannin Krysiak, Joshua F McMichael, Adam C Coffman, Arpad M Danos, Benjamin J Ainscough, Cody A Ramirez, Damian T Rieke, Lynzey Kujan, Erica K Barnell, Alex H Wagner, Zachary L Skidmore, Amber Wollam, Connor J Liu, Martin R Jones, Rachel L Bilski, Robert Lesurf, Yan-Yang Feng, Nakul M Shah, Melika Bonakdar, Lee Trani, Matthew Matlock, Avinash Ramu, Katie M Campbell, Gregory C Spies, Aaron P Graubert, Karthik Gangavarapu, James M Eldred, David E Larson, Jason R Walker, Benjamin M Good, Chunlei Wu, Andrew I Su, Rodrigo Dienstmann, Adam A Margolin, David Tamborero, Nuria Lopez-Bigas, Steven J M Jones, Ron Bose, David H Spencer, Lukas D Wartman, Richard K Wilson, Elaine R Mardis, Obi L Griffith

**Affiliations:** 1 McDonnell Genome Institute, Washington University School of Medicine, St. Louis, Missouri, USA; 2 Siteman Cancer Center, Washington University School of Medicine, St. Louis, Missouri, USA; 3 Department of Genetics, Washington University School of Medicine, St. Louis, Missouri, USA; 4 Department of Medicine, Washington University School of Medicine, St. Louis, Missouri, USA; 5 Charité Comprehensive Cancer Center, Charité Universitätsmedizin, Berlin, Germany; 6 Michael Smith Genome Sciences Centre, British Columbia Cancer Agency, Vancouver, British Columbia, Canada; 7 Department of Molecular and Experimental Medicine, Scripps Research Institute, La Jolla, California, USA; 8 Oncology Data Science Group, Vall d’Hebron Institute of Oncology, Barcelona, Spain; 9 Computational Biology, Oregon Health and Science University, Portland, Oregon, USA; 10 Institute for Research in Biomedicine, Barcelona Institute of Science and Technology, Barcelona, Spain

## Abstract

CIViC is an expert-crowdsourced knowledgebase for Clinical Interpretation of Variants in Cancer describing the therapeutic, prognostic, diagnostic and predisposing relevance of inherited and somatic variants of all types. CIViC is committed to open-source code, open-access content, public application programming interfaces (APIs) and provenance of supporting evidence to allow for the transparent creation of current and accurate variant interpretations for use in cancer precision medicine.

Understanding of the genetic heterogeneity and mutational landscape underlying cancer has seen incredible advances in recent years. This has accelerated the implementation of precision medicine strategies in which clinicians and researchers target specific molecular variants with treatments tailored to the individual and their disease^1^. The biomedical literature describing such associations is large and growing rapidly. As a result, the interpretation of individual variants observed in patients has become a bottleneck in clinical sequencing workflows^2^. Many cancer hospitals and research centers are engaged in separate efforts to interpret cancer-driving variants and genes in the context of clinical relevance. These efforts are largely occurring within independent ‘information silos’, producing interpretations that require constant updates, lack community consensus and involve intense manual input.

Estimates of the proportion of patients with cancer who would benefit from comprehensive molecular profiling vary substantially^3^, in part because of the lack of both a community consensus definition of actionability and a comprehensive catalog of specific clinical variant interpretations. Achieving the goals of precision medicine will require this information to be centralized, freely accessible, openly debated and accurately interpreted for application in the clinic. Existing efforts to facilitate clinical interpretation of variants include the Gene Drug Knowledge Database^4^, the Database of Curated Mutations^5^, ClinVar^6^, ClinGen^7^, PharmGKB^8^, Cancer Driver Log^9^, My Cancer Genome^10^, Jax-Clinical Knowledgebase^11^, the Personalized Cancer Therapy Knowledgebase, the Precision Medicine Knowledgebase, the Cancer Genome Interpreter, OncoKB and others (Supplementary Table 1). These resources often have barriers to widespread adoption, including some combination of (i) no public access to content, (ii) restrictive content licenses, (iii) no public API, (iv) no bulk data download capabilities and (v) no mechanism for rapid improvement of the content (see Supplementary Table 1 for detailed feature comparison). To address these limitations, we present CIViC, an open-access, open-source knowledgebase for expert crowdsourcing of Clinical Interpretation of Variants in Cancer (http://civicdb.org/; Fig. 1).

The critical distinguishing features of the CIViC initiative, in comparison to several of the resources cited above, stem from its strong commitment to openness and transparency. We believe that these principles (Box 1) are necessary for widespread adoption of such a resource. The target audience of CIViC is deliberately broad, encompassing researchers, clinicians and patient advocates. CIViC is designed to encourage development of community consensus by leveraging an interdisciplinary, international team of experts collaborating remotely within a centralized curation interface. Variant interpretations are created with a high degree of transparency and detailed provenance. The interface is designed to help keep interpretations current and comprehensive, and to acknowledge the efforts of content creators (Supplementary Fig. 1). CIViC accepts public knowledge contributions but requires that experts review these submissions.

Box 1 **CIViC principles**

1. **Interdisciplinary.** An interdisciplinary approach is needed to combine the expertise of genome scientists, healthcare providers, patient advocates and others.
2. **Community consensus.** The interpretations of clinical actionability required to enable precision medicine should be freely available and openly discussed across a diverse community. To facilitate consensus building, the interface must support direct contribution from members of the community.
3. **Transparency.** Content should be created with transparency, kept current, be comprehensive, track provenance and acknowledge the efforts of its creators.
4. **Computationally accessible.** The interface should be both structured enough to allow computational data mining (via APIs) and agile enough to handle the product of openly debated human interpretation.
5. **Freely accessible.** Curated knowledge will remain free and can be accessed anonymously without login unless the user wishes to contribute to content. No fees will be introduced.
6. **Open license.** CIViC will encourage both academic and commercial engagement through flexible licensing. Access will not be restricted by exclusive licensing.

The manner in which the clinical relevance of variants in cancer is presented in the published literature is highly heterogeneous. To represent these data in a more easily interpretable and consistent fashion, the CIViC data model is highly structured and ontology driven (Supplementary Fig. 2). Clinical interpretations are captured and displayed as evidence records consisting of a freeform ‘evidence statement’ and several structured attributes. Each evidence record is associated with a specific gene, variant, disease and clinical action. Evidence records belong to one of four evidence types indicating whether a variant is predictive of response to therapy, prognostic, diagnostic and/or predisposing for cancer. Evidence records are also assigned to an evidence level ranging from established clinical utility (level A) to inferential (level E) evidence (Supplementary Figs. 3 and 4). The quality of the underlying published evidence is rated from one to five stars. As evidence records accumulate for a single variant, they are in turn synthesized into a human-readable ‘variant summary’ of the variant’s overall significance in cancer. Variants can also be aggregated into ‘variant groups’ that share a clinical significance (for example, imatinib resistance). All variant types are supported (including structural variants, RNA fusions and other expression events) as well as all variant origins (somatic mutation, germline mutation and germline polymorphism). Genomic coordinates, transcript identifiers and variant synonyms are determined by curators, reviewed by editors and stored in a standardized format (for example, HGVS) for each variant. Additional variant information is imported through the MyVariant.info annotation API^12^, providing links to complementary resources and variant annotations such as ClinVar^6^, COSMIC^13^ and ExAC^14^. Each variant is associated with a single gene, and each gene view provides a curated ‘gene summary’ synthesizing all of the variants it contains. Additional gene information is imported through the MyGene.info annotation API^12^, allowing users to focus curation effort on clinical impact and not repeat the efforts of other databases. Integration of public ontologies and databases, such as Disease Ontology^15^ and Sequence Ontology^16^, allows CIViC’s data to be formally structured (Supplementary Fig. 5) and integrated with other resources. This structure provides both computationally accessible information and human-interpretable content with the flexibility to capture key details for the wide range of variants and experiment types being interpreted (refer to the Supplementary Note for implementation details).

CIViC currently contains 1,678 curated interpretations of clinical relevance for 713 variants affecting 283 genes (Supplementary Fig. 6). These interpretations were curated from 1,077 published studies by 58 CIViC curators. CIViC evidence records are supported by a wide range of evidence levels and trust ratings, currently biased toward somatic alterations and positive associations with treatment response (Supplementary Fig. 7). At least one evidence record has been created for 209 cancer subtypes and 291 drugs, with some bias toward lung, breast, hematologic, colorectal and skin cancers and associated targeted therapies (Supplementary Fig. 8). Supporting publications for these interpretations come from a large number of journals, primarily over the last five years, and tend to provide just one or two evidence records each (Supplementary Fig. 9). From the public launch of CIViC in June 2015 to December 2016, external curators (not affiliated with Washington University) contributed 46.7% of the evidence statements within the knowledgebase (Supplementary Fig. 6b). Thus far, submissions, revisions, comments and expert reviews have produced 11,254 distinct curation actions. These numbers continue to grow. More than 16,000 users have accessed CIViC interpretations from academic, governmental and commercial institutions around the world (Supplementary Fig. 6c,d). Early adopters of CIViC include leaders in developing cancer genomics pipelines^17^, the UCSC Genome Browser^18^ and Agilent’s Cartagenia Bench Lab NGS. Early curation and content partners include the Gene Drug Knowledgebase^4^ and the Personalized Oncogenomics Program^19^. The CIViC resource is freely accessible without login, and no fees or exclusive access will be introduced in the future. Both academic and commercial adoption is free and encouraged. The variant and gene summaries, with additional statistics summarizing the level of supporting evidence in CIViC, can be automatically incorporated into clinical reports using the CIViC API or bulk data releases (updated nightly, with stable monthly releases) (Fig. 1). The source code for the CIViC website and public API are released under an open-source license (the Massachusetts Institute of Technology or ‘MIT’ license), and all curated content within CIViC is released under an open-access license (the Creative Commons Public Domain Dedication or ‘CC0’ license). The unencumbered availability of the CIViC bulk data releases, lack of requirements to establish a licensing agreement, the well-documented public API, and use of a structured data model and ontologies allow rapid adoption of CIViC in clinical workflows. As the user base grows, the number of experts with a vested interest in the content will increase, driving community engagement and increasing curation from external users.

A critical concern, as CIViC content expands, is the maintenance of high-quality data and the inherent tradeoff between data quality and rapid or automated updating. The curation workflow of CIViC (Supplementary Fig. 10) requires agreement between at least two independent contributors before acceptance of new evidence or revisions of existing content (Supplementary Figs. 11 and 12). At least one of these users must be an expert editor, and editors are barred from approving their own contributions. CIViC includes features such as typeahead suggestions (recommendations that appear as soon as you start to type), automatic warning of possible duplicates, detailed documentation in all entry forms, and input validation to encourage high-quality data entry. To facilitate team curation efforts (Supplementary Fig. 10), the CIViC interface also includes features such as subscriptions, notifications and mentions. Curators can also use an advanced search interface to generate and share complex queries of CIViC data that help guide curation effort and content consumption (Supplementary Fig. 13). Many of these features were inspired by the ‘best practices’ of active online collaborative research and software development platforms, including BioStars^20^ and GitHub.

A major challenge to the success of CIViC is the scope and complexity of the knowledge that needs to be summarized, and the development of strategies to assess the completeness of the resource. The American College of Medical Genetics and Genomics (ACMG) and the Association for Molecular Pathology (AMP) recently reported on the variability among nine laboratories in clinical interpretations of germline variants relevant to Mendelian diseases^21^, a field where the ACMG–AMP have proposed detailed standards and guidelines for variant classification^22^. This report identified a low rate of interpretation agreement between laboratories (34% concordance). However, discussion and review of criteria were able to more than double this concordance, demonstrating the need for and success of open discourse in clinical variant interpretation^21^. Recently, the Somatic Working Group (WG) of the Clinical Genome Resource (ClinGen) has published a consensus set of minimal variant-level data (MVLD) to help standardize data elements needed for curation of the clinical utility of somatic cancer variants^23^. At present, cancer variant interpretation efforts that nominally have the same goals show a remarkably low overlap in source publications cited for these interpretations (1.6–71.6%, but generally less than 25%; Supplementary Table 2). This suggests that no single effort has comprehensively identified or summarized even the most relevant literature in this area, further illustrating the high curation burden involved. Conversely, these small overlaps emphasize the importance of reducing duplication of effort moving forward, especially considering the vastness of the existing literature and its tremendous growth rate. In CIViC, curation efforts thus far have focused on variants relevant to cancer types of particular interest at our center (for example, acute myeloid leukemia, breast cancer and lung cancer; Supplementary Fig. 8b), on variants identified as high priority by early CIViC partners^4,19^ and on variants targeted by proof-of-principle precision medicine ‘basket’ clinical trials such as NCI-MATCH (also known as EAY131 or NCT02465060). Our ability to provide expertise in these areas is complemented by the expert knowledge of other groups and organizations, making CIViC a more comprehensive resource than would be possible with a ‘siloed data’ approach. To this end, recruitment of external contributors and domain experts from multiple fields is a top priority. This is accomplished in part through planning of CIViC-sponsored events in the cancer research and treatment community. We also allow for different levels of external community involvement, including submission of suggested publications to a queue to guide others to generate new evidence records (Supplementary Fig. 14).

Additional challenges faced by CIViC include long-term sustainability of funding, ensuring broad clinical engagement and maintaining the enthusiasm for the crowd-sourcing efforts. We are addressing each of these challenges by engagement with other resources, experts and funding agencies with track records of long-term maintenance of informatics resources (see the Supplementary Note for further discussion). To facilitate such engagement and seek broad input and external guidance for our efforts, we have recently established a Variant Interpretation for Cancer Consortium (VICC) as part of the Global Alliance for Genomics Health (GA4GH). We have also established a panel of clinical domain experts to provide independent guidance on development of the resource and to assess the completeness and accuracy of our variant curation efforts.

CIViC is designed to address many of the challenges of cancer variant interpretation. To our knowledge, CIViC is the only variant interpretation effort currently capable of leveraging community experts and additionally has the most open model (open-access content, open-source code and an open API). We believe that this open strategy represents a sustainable model for achieving current, standardized and comprehensive interpretations of the clinical relevance of cancer variants. As the community of contributors grows, an increased incentive will emerge to help keep CIViC updated with cutting-edge clinical trial and US Food and Drug Administration (FDA) investigational new drug (IND) findings. As we have created a comprehensive and modern API, centers can rapidly integrate CIViC into automated clinical report generation for gene panel, exome, whole-genome and RNA sequencing of tumor samples. While there are many challenges faced by an effort such as this one, we hope that, with input from a critical mass of interested parties, these challenges can be largely overcome. We invite all researchers, healthcare providers and patient advocates engaged in clinical interpretation of variants to join the community at CIViC (http://civicdb.org/).

## URLs

The Clinical Interpretation of Variants in Cancer resource described by this work is available at http://www.civicdb.org/. Personalized Cancer Therapy Knowledgebase, https://pct.mdanderson.org/; Precision Medicine Knowledgebase, https://pmkb.weill.cornell.edu/; Cancer Genome Interpreter, https://www.cancergenomeinterpreter.org/; OncoKB, http://oncokb.org/; GitHub, https://github.com/; GA4GH Variant Interpretation for Cancer Consortium (VICC), http://ga4gh.org/#/vicc; MIT license, https://open-source.org/licenses/MIT; CC0 license, https://creativecommons.org/publicdomain/zero/1.0/.

## Supplementary Material

Supplementary Materials

## Figures and Tables

**Figure 1:**
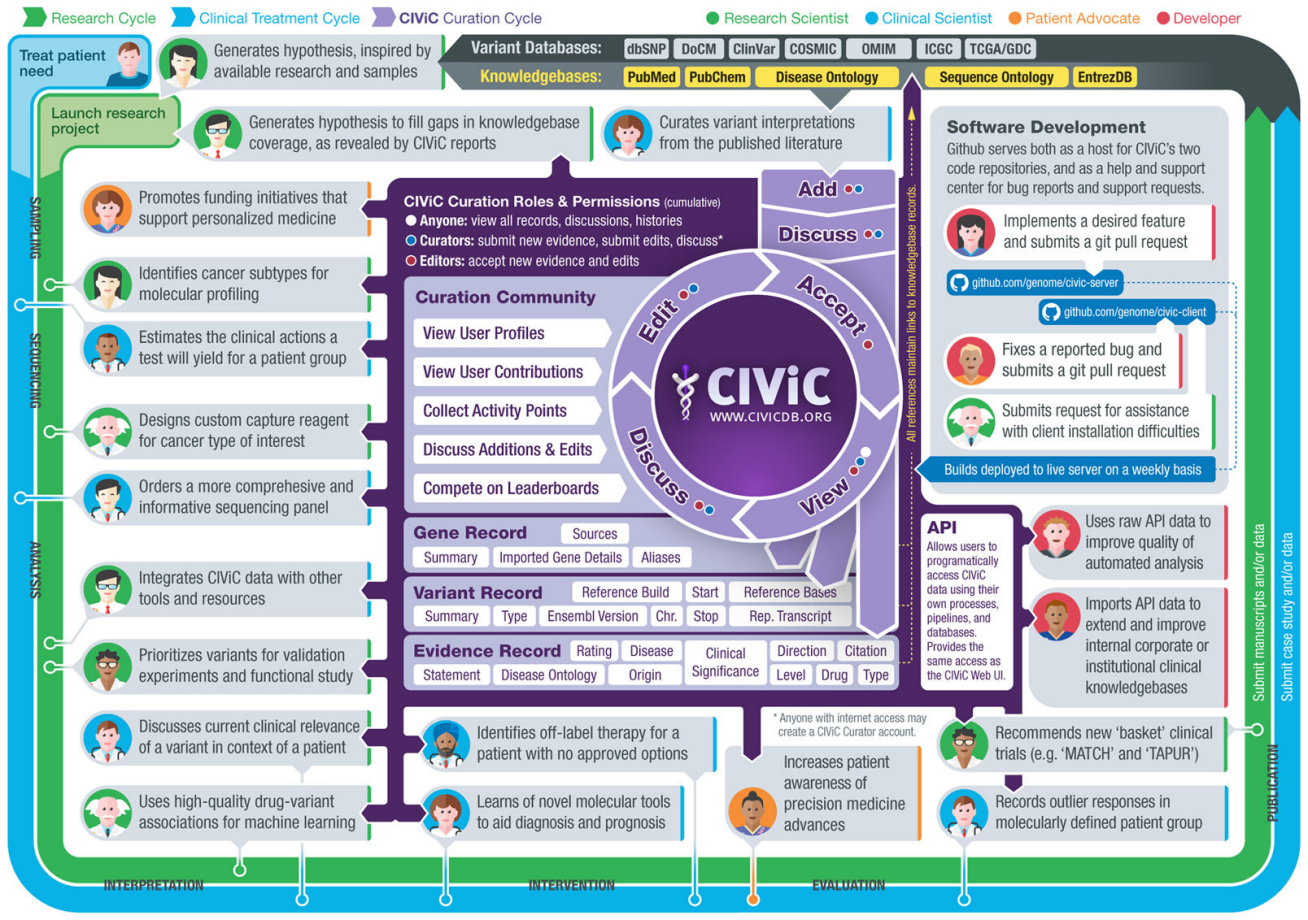
Contribution of CIViC to the precision cancer treatment cycle. The diagram summarizes how research, clinical treatment and CIViC knowledge curation are interrelated. The CIViC knowledgebase aims to develop clinical interpretations for raw cancer variant observations stored in large variant databases (gray). Each CIViC variant interpretation is based on published evidence and leverages complementary knowledge bases and ontologies wherever possible (yellow). The precision medicine clinical treatment cycle (blue) and research cycle (green) both involve sampling, sequencing, analysis, interpretation, intervention, evaluation and publication. These cycles start with hypothesis generation, followed by research projects or clinical trials, and dissemination of their findings. Examples of how each stage specifically relates to or benefits from the CIViC resource are represented by ‘persona’ icons for the four types of CIViC stakeholders: research scientists (green), clinical scientists (blue), patient advocates (orange) and developers (red). Each is accompanied by a brief description of a possible research, clinical, outreach or software development action. In the center of the diagram, key features of the CIViC interface and data model are summarized (purple). These include the roles and permissions of CIViC users, especially consumers of the content, curators and editors. Members of the CIViC community participate by adding, editing, discussing and approving individual evidence records and summaries that support the clinical interpretation of cancer variants. Anyone willing to log in may assume the role of curator, but contributions must be reviewed by expert editors before acceptance.

## References

[1] Collins FS, Varmus H (2015). A new initiative on precision medicine. N Engl J Med.

[2] Good BM, Ainscough BJ, McMichael JF, Su AI, Griffith OL (2014). Organizing knowledge to enable personalization of medicine in cancer. Genome Biol.

[3] Meric-Bernstam F (2015). Feasibility of large-scale genomic testing to facilitate enrollment onto genomically matched clinical trials. J Clin Oncol.

[4] Dienstmann R, Jang IS, Bot B, Friend S, Guinney J (2015). Database of genomic biomarkers for cancer drugs and clinical targetability in solid tumors. Cancer Discov.

[5] Ainscough BJ (2016). DoCM: a database of curated mutations in cancer. Nat Methods.

[6] Landrum MJ (2016). ClinVar: public archive of interpretations of clinically relevant variants. Nucleic Acids Res.

[7] Rehm HL (2015). ClinGen—the Clinical Genome Resource. N Engl J Med.

[8] Thorn CF, Klein TE, Altman RB (2013). PharmGKB: the Pharmacogenomics Knowledge Base. Methods Mol Biol.

[9] Damodaran S (2015). Cancer Driver Log (CanDL): catalog of potentially actionable cancer mutations. J Mol Diagn.

[10] Yeh P (2013). DNA-Mutation Inventory to Refine and Enhance Cancer Treatment (DIRECT): a catalog of clinically relevant cancer mutations to enable genome-directed anticancer therapy. Clin Cancer Res.

[11] Patterson SE (2016). The clinical trial landscape in oncology and connectivity of somatic mutational profiles to targeted therapies. Hum Genomics.

[12] Xin J (2016). High-performance web services for querying gene and variant annotation. Genome Biol.

[13] Forbes SA (2017). COSMIC: somatic cancer genetics at high-resolution. Nucleic Acids Res.

[14] Karczewski KJ (2017). The ExAC browser: displaying reference data information from over 60 000 exomes. Nucleic Acids Res.

[15] Kibbe WA (2015). Disease Ontology 2015 update: an expanded and updated database of human diseases for linking biomedical knowledge through disease data. Nucleic Acids Res.

[16] Eilbeck K (2005). The Sequence Ontology: a tool for the unification of genome annotations. Genome Biol.

[17] Gagan J, Van Allen EM (2015). Next-generation sequencing to guide cancer therapy. Genome Med.

[18] Speir ML (2016). The UCSC Genome Browser database: 2016 update. Nucleic Acids Res.

[19] Laskin J (2015). Lessons learned from the application of whole-genome analysis to the treatment of patients with advanced cancers. Cold Spring Harb Mol Case Stud.

[20] Parnell LD (2011). BioStar: an online question & answer resource for the bioinformatics community. PLOS Comput Biol.

[21] Amendola LM (2016). Performance of ACMG-AMP Variant-Interpretation Guidelines among nine laboratories in the Clinical Sequencing Exploratory Research Consortium. Am J Hum Genet.

[22] Richards S (2015). Standards and guidelines for the interpretation of sequence variants: a joint consensus recommendation of the American College of Medical Genetics and Genomics and the Association for Molecular Pathology. Genet Med.

[23] Ritter DI (2016). Somatic cancer variant curation and harmonization through consensus minimum variant level data. Genome Med.

