# Supplementary material for: CIViC is a community knowledgebase for expert crowdsourcing the clinical interpretation of variants in cancer
Source: Nat Genet. Author manuscript; Available in PMC 2017 Mar 27. (PMC5367263; doi:10.1038/ng.3774)
Supplement: Supplementary Materials [file NIHMS851295-supplement-Supplementary_Materials.pdf]

Supplementary Materials

CIViC is a community knowledgebase for expert-crowdsourcing the clinical interpretation of variants in cancer

Malachi Griffith[1,2,3,4,*], Nicholas C Spies[1,*], Kilannin Krysiak[1,4,*], Joshua F McMichael[1], Adam C Coffman[1], Arpad M Danos[1], Benjamin J Ainscough[1,2,3], Cody A Ramirez[1], Damian T Rieke[5], Lynzey Kujan[1], Erica K Barnell[1], Alex H Wagner[1,2], Zachary L Skidmore[1], Amber Wollam[1], Connor J Liu[1], Martin R Jones[6], Rachel L Bilski[1], Robert Lesurf[1], Yan-Yang Feng[1], Nakul M Shah[1], Melika Bonakdar[6], Lee Trani[1], Matthew Matlock[1], Avinash Ramu[1], Katie M Campbell[1], Gregory C Spies[1], Aaron P Graubert[1], Karthik Gangavarapu[7], James M Eldred[1], David E Larson[1,3], Jason R Walker[1], Benjamin M Good[7], Chunlei Wu[7], Andrew I Su[7], Rodrigo Dienstmann[8], Adam A Margolin[9], David Tamborero[10], Nuria Lopez-Bigas[10], Steven JM Jones[6], Ron Bose[4], David H Spencer[1,4], Lukas D Wartman[1,2,4], Richard K Wilson[1,2,3,4], Elaine R Mardis[1,2,3,4], Obi L Griffith[1,2,3,4]

[1] McDonnell Genome Institute, Washington University School of Medicine, St. Louis, MO, USA;
[2] Siteman Cancer Center, Washington University School of Medicine, St. Louis, MO, USA;
[3] Department of Genetics, Washington University School of Medicine, St. Louis, MO, USA;
[4] Department of Medicine, Washington University School of Medicine, St. Louis, MO, USA;
[5] Charité Comprehensive Cancer Center, Charité – Universitätsmedizin, Berlin, Germany;
[6] Michael Smith Genome Sciences Centre, British Columbia Cancer Agency, Vancouver, BC, Canada;
[7] Department of Molecular and Experimental Medicine, The Scripps Research Institute, La Jolla, CA, USA;
[8] Oncology Data Science Group, Vall d'Hebron Institute of Oncology, Barcelona, Spain;
[9] Computational Biology, Oregon Health and Science University, Portland, OR, USA;
[10] Institute for Research in Biomedicine, The Barcelona Institute of Science and Technology, Barcelona, Spain.

*These authors contributed equally to this work.

Correspondence should be addressed to MG (mgriffit@wustl.edu) and OLG (obigriffith@wustl.edu).

Supplementary Note

Implementation Details

The CIViC source code and application are organized in a client-server model. The code is developed using a continuous integration and test-driven approach. The server side consists of a Ruby/Rails web application that interacts with a PostgreSQL relational database (**Supplementary Figure 5**). The server provides JSON API endpoints to the client. User authentication is managed by ‘Oauth v2’ and currently supports login with a user’s existing ORCID, GitHub, or Google account. ‘Code Climate’ is used to evaluate code quality, ‘Travis CI’ for automated code testing, and ‘Coveralls’ to evaluate test coverage (currently 92%). The client side consists of an ‘AngularJS’ application that interacts with the CIViC server. It uses ‘NPM’ and ‘Bower’ for package management, and ‘Gulp’ to build the JavaScript application. Code changes are first pushed to a staging server for testing before being deployed to the public server using ‘Puppet’. Current development efforts can be followed in the public GitHub pages at https://github.com/genome/civic-client (front end) and https://github.com/genome/civic-server (back end). Anyone is free to submit pull requests or issues (feature requests, bug reports, etc.) to these repositories. Using cutting edge methods and software development best-practices promotes integration with future end-user development and implementation tasks with incentive for developers to improve the underlying CIViC resource.

Data Availability

All data created by the CIViC project are freely available under an open access creative commons public domain attribution (CC0) at https://civic.genome.wustl.edu/ (or http://www.civicdb.org).

Code Availability

All code used by the CIViC project are freely available under an open source license (MIT) at https://github.com/genome/civic-server/ and https://github.com/genome/civic-client/. The CIViC code is maintained using the version control system ‘git’ (https://git-scm.com/).

Sustainability of the CIViC project

Long term sustainability is an important challenge to address in resources such as these. The CIViC project was initiated approximately three years ago, and the first beta version of the interface went online almost two years ago. There are several factors that support our claims of long term sustainability. First, we have considerable institutional support from Washington University. Second, we have a solid track record of supporting online resources. For example, a previous resource, www.dgidb.org has been online for well over four years and is still being actively developed with an update paper recently published[1] and another in preparation. Third, there appears to be an encouraging trend among funding agencies to recognize the importance of funding the creation, development, and maintenance of informatics resources. For example, to further develop CIViC we were awarded funding under a program for “Early-Stage Development of Informatics Technologies for Cancer Research and Management (U01)” (PAR-15-332). The NCI also currently offers awards for “Sustained Support for Informatics Resources for Cancer Research and Management (U24)” (PAR-15-333). This program is accepting applications until the end of 2018. If we are able to demonstrate CIViC as a valuable resource to the cancer research community, this mechanism could, in theory, support the resource until Spring 2024. Alternatively, additional new promising mechanisms are available from NHGRI, NLM, NIGMS, etc. To support hosting/compute costs we intend to apply for supplemental funding in the form of cloud computing credits (up to $50,000) through the recently announced NIH Commons Credit Portal (https://www.commons-credit-portal.org/). Additional hosting grants for research projects are available directly from cloud providers including Google Cloud, Microsoft Azure, and Amazon AWS. The relatively modest bandwidth and storage requirements of a knowledgebase like CIViC translates into modest hosting costs, especially compared to resources that rely on raw genomic sequence data. Fourth, we are working closely with

the Global Alliance for Genomics Health (GA4GH) to identify long term support options for variant interpretation efforts worldwide (including CIViC). Fifth, it may be possible to partner with commercial entities without sacrificing the open principles of CIViC such as universal public access. It bears repeating that these principles are fundamental to the project and would not be altered for such commercial support.

In addition to all of the above strategies (and more) that we will pursue to ensure the long term sustainability, growth, and improvement of CIViC, we will also seek advice and guidance from the creators of exemplar resources that have demonstrated wide community adoption and long term maintenance. For example, we will seek input from projects such as the Ensembl Genome Browser (online since at least 2000), the UCSC genome browser (2000-present), DrugBank (2006-present), PharmGKB (2000-present), IGV (2008-present), Bioconductor (2001-present), etc. To supplement the team of domain experts and clinicians we have assembled to improve the clinical relevance of CIViC, we will create an informal panel of informatics resource experts to help establish a sustainable long term development and maintenance roadmap for CIViC. Finally, CIViC is a completely open source project. Others can fork the entire project and all curated data without any legal encumbrance. Anyone can contribute code in the same way that they can contribute curation effort. This approach reduces the risk of CIViC disappearing completely as it allows for a decentralized maintenance model that should be more robust.

Quality assurance

To ensure quality of the interpretations created in CIViC, mechanisms to enable external assessment are of clear importance. There are several concrete mechanisms we plan to employ to engage knowledgeable external reviewers. Several of these are already under way. First, we recognize that many users will find CIViC lacking variants which they know to have established prognostic, predictive, diagnostic or predisposing value, and that these users might not have the time to immediately curate these evidence statements themselves. To address this issue, a new publication or “source suggestion” queue has been added to the CIViC web interface (**Supplemental Figure 14**). This new feature allows external experts to quickly and easily add important publications (using PubMed ID) to a queue for later generation of CIViC evidence records by the curation team. In addition to PubMed ID, an entry to the queue contains a free text field whereby publication submitters can add comments to help guide curation efforts. Optional fields available when creating an entry for the publication queue are gene, variant, and disease. A second mechanism for assessing the completeness of CIViC content is the recruitment of external domain experts to join the CIViC network and assess the resource in their respective areas of expertise. Thus far, we have reached out to 30 authors whose publications appear in CIViC to review curation that has been done in their area of expertise. We will continue to use measures such as these to identify additional highly-relevant external experts. Third, a portion of existing CIViC grant funds are dedicated to hosting events engaging the scientific community. The first of these events consisted of a multi-day hackathon and curation jamboree at the Netherlands Cancer Institute in Amsterdam (https://civic.genome.wustl.edu/#/meetings). At this event, we continued our recruitment of external domain experts to assess CIViC content and add publications to the variant queue.

While the above gives concrete examples of ways to engage experts for external validation, there is also a need to create objective approaches to assess comprehensiveness that are independent of interested parties. One such approach is comparison of CIViC content to other databases (such a comparison is described in **Supplementary Table 2** of the manuscript). This will ensure that CIViC is consistent with the existing literature and up to date with other curation efforts. Another method we use to objectively identify gaps within the database is to actively seek out lists of variants used in cancer capture reagents (made public here: https://civic.genome.wustl.edu/#/help/evidence). This allows CIViC curators to identify variants in those lists that have published prognostic, diagnostic, predictive, or predisposing value to ensure that the CIViC database becomes a reflection of the current state of knowledge. Also, internally generated statistics regarding CIViC coverage of variant-phenotype associations (e.g., diseases, drugs; **Supplementary Figures 8-9**) can be directly compared to similar statistics appearing for instance in reviews or competing resources, giving a further

external measure of completeness. Finally, we have recently initiated a collaboration that will use natural language processing methods to automatically mine the literature for evidence that should be reviewed by curators for inclusion in CIViC.

Clinical engagement

Throughout the development of CIViC, we have sought input from clinical collaborators and have developed several mechanisms for more formal engagement. Currently, one-third of our “domain experts” are physician scientists. This resource was born out of our own needs based on collaborations with clinicians and experiences with data analysis for Washington University’s Genomics Tumor Board. The development has included regular discussions with and presentations to these collaborators in addition to our domain experts.

The underlying mission of CIViC is to curate actionable variants in cancer into clinician accessible summaries. This type of resource has obvious clinical utility. First, it has the potential to summarize multiple trials and case reports into a concise comprehensive report thereby increasing the power of each statement that is curated. Second, it provides a user-friendly interface for all cancer variants, which will reduce the time required for physicians to understand the actionable variants associated with individual patients. Finally, the open crowdsourced nature of the resource allows for continuous, dynamic updates to ensure that statements are more likely to be truly representative of the existing science. We recognize however, that this vision for a one-stop-shop for cancer variants has not yet been realized. While many variants and diseases are covered with significant depth, many are not. To address this issue, we are constantly seeking to incorporate stronger clinical links through a variety of means. Specifically, we have engaged clinical domain experts who will review changes relevant to their field of expertise and give their stamp of approval on finalized summaries. We have also met with a number of pathology groups and services regarding their use of the resource, and are incorporating their feedback. We also recognize that the database, in its current state, will act as a supplementary resource for physicians to better understand their patients’ variants and potential therapies that could be used for these individuals. CIViC has just recently been implemented as such a supplementary resource to be provided to users of Agilent’s Cartegenia resource which, through discussions with Agilent developers, will provide insight into the utility of CIViC to Cartegenia users.

Prior to launching the database as a method to direct patient protocols in a CLIA setting (which could be potentially harmful to patients) we anticipate putting the database through a rigorous evidence-based trial to understand the benefits and drawbacks. This will require validating a CIViC informed capture panel and associated variant interpretations in a clinical setting. The end goal of such a trial is a bench-to-bedside patient report on the interpretation of any patient’s variants for use by their physician.

Maintaining enthusiasm of crowdsourcing

To encourage ongoing and sustainable engagement, we have formed a new working group of the Global Alliance for Genomics Health (GA4GH) called the Variant Interpretation for Cancer Consortium (VICC; http://ga4gh.org/#/vicc). The goals of this group are to (1) harmonize global efforts for clinical interpretation of cancer variants by forming an open consortium of developers and curators committed to eliminating the interpretation bottlenecks for precision medicine in cancer and (2) implement software systems to query across standardized knowledgebases. In support of this effort we have created an agreement of data sharing principles. We have also identified a consortium funding opportunity that could specifically support active engagement between competing resources in this area. The first VICC conference call was attended by 50+ representatives from almost all centers with competing databases (MSKCC, OHSU, Dana Farber, MD Anderson, Illumina, Weill Cornell, Princess Margaret Hospital, etc.). Discussion on how to promote ongoing engagement and cooperation was continued at the 4th GA4GH plenary meeting in Vancouver. Another major goal of this meeting was to explore how the CIViC project can best interface with the highly relevant Cancer Gene Trust and ClinGen initiatives. We also have ongoing discussions with relevant clinical initiatives including the NCI Match Clinical Trial and ASCO’s CancerLinq. We have also discussed CIViC with many commercial

organizations that may benefit from it. We will continue to present updates regularly at relevant conferences, promote the resource on social media, etc. In other words, we are active in engaging with a broad cross-section of the cancer genomics community. We hope that this ongoing engagement along with the very open model of CIViC will encourage widespread use of CIViC not just by individual users but by other competing databases as well. This widespread adoption will in turn help to encourage engagement with crowdsourcing participants. The more widely and comprehensively used CIViC is, the more likely it is that users will begin to make incremental contributions.

Integration with ClinVar and ClinGen efforts

ClinVar and ClinGen are excellent resources and are utilized extensively by CIViC curators to create evidence statements. Specifically, we are working with ClinGen to ensure CIViC's compliance with their recently defined Minimum Variant Level Data (MVLD) guidelines[2]. Representatives from ClinGen attended our National Cancer Institute funded Curation Jamboree and Hackathon at the Netherlands Cancer Institute (NKI) (https://civic.genome.wustl.edu/#/meetings) and helped to improve the interface, curation coverage, and community engagement. The ClinVar, ClinGen and CIViC groups are interested in cross-pollination between these resources. With respect to ClinVar, we have realized that bulk import of all information within this database might cause problems due to the lack of reviewed evidence supporting some variants. However, ClinVar records with 3-star or 4-star status have high priority for curation in CIViC. We hope to use ClinVar to help populate the newly added curation queue (described above) within the CIViC database with high impact variants and sources. We also agree that leveraging existing rich information in ClinVar and other resources is important. In response to this suggestion we have added a new feature to CIViC that uses the MyVariant.info API[3] to automatically integrate CIViC variants with extensive external information on each variant from ClinVar, COSMIC, and other key resources. Through this method, 73 variants in CIViC have now been linked to corresponding ClinVar records, 11 of which have 3-stars. Regardless of whether ClinVar records are matched via MyVariant.info, ClinVar record IDs can now be directly linked at the variant level as shown in **Supplementary Figures 1 and 11**. We are also considering a new feature that would allow a ClinVar variant record itself to act as an evidence source so that we can capture pathogenic variants with strong support from multiple laboratories that nevertheless might never be published in a PubMed indexed peer-reviewed article.

Supplementary Figures

Supplementary Figure 1. CIViC interface overview

The user-friendly CIViC interface is the primary point of contact with users whether they are consuming, editing or adding content. CIViC user-curated content (blue boxes) is visible without sign in and provides the bulk of visible content ordered from gene level (top) to variant level (middle), and finally individual evidence records (bottom). Curated content is enhanced by imported content and citations (orange boxes) that are linked directly to their original source. Website navigation and extensive documentation are highlighted with red boxes. Finally, a curator can interact (green boxes) with CIViC user-curated content by 1) suggesting changes (edit button) or adding content; 2) commenting on content or suggested revisions; 3) downloading content; or 4) viewing their activity, suggested changes, notifications, or profile.

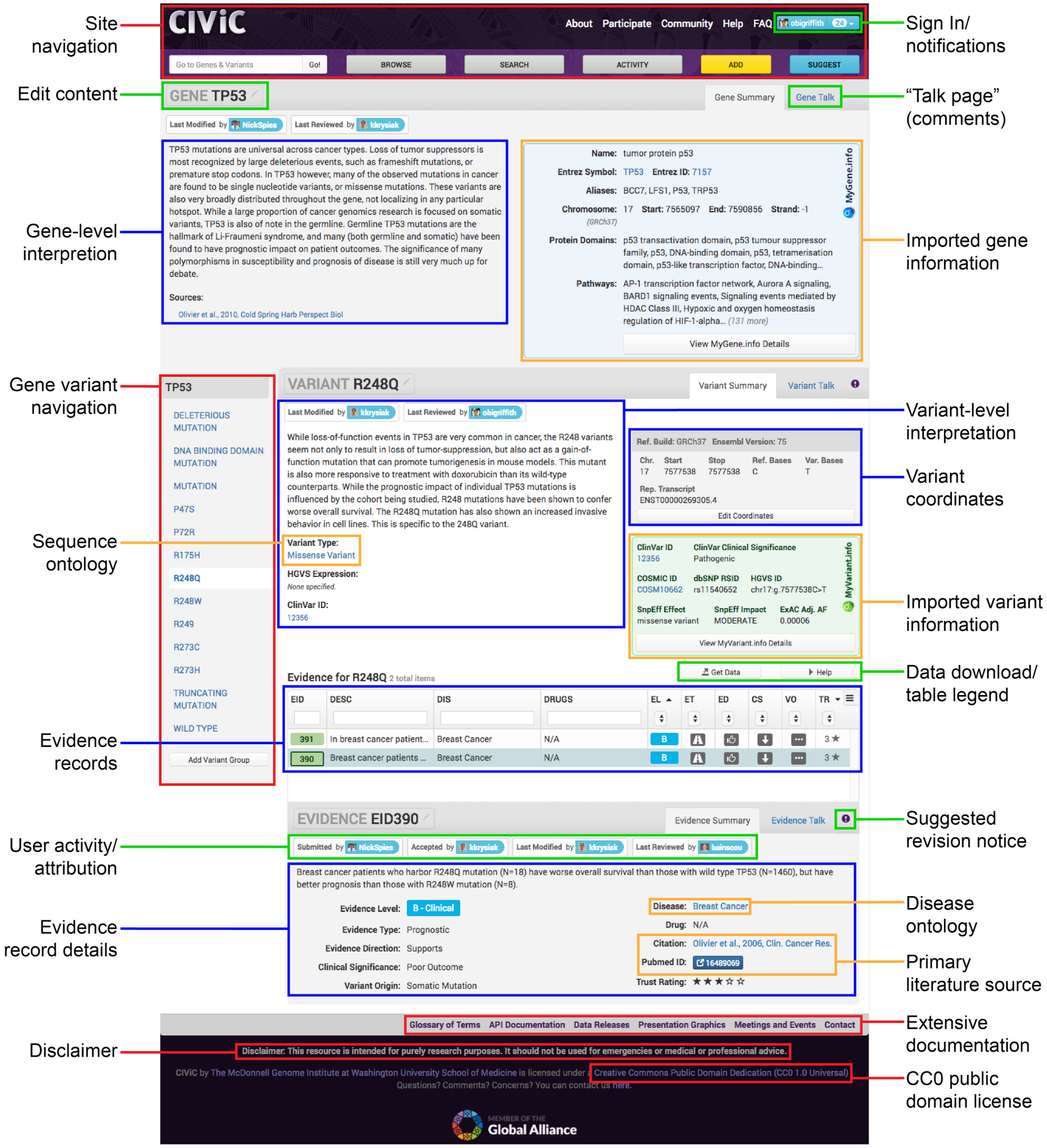

Supplementary Figure 2. The CIViC data model
Key elements of the CIViC data model are listed below. Briefly, CIViC aims to provide gene and variant level executive summaries of the clinical relevance of specific variants. Multiple structured evidence records are first created and then synthesized to produce these executive variant/gene summaries. Each evidence record is associated with a specific variant and gene. Each evidence record also corresponds to a single clinical assertion for a single cancer type from a single peer-reviewed publication. One publication can be used to generate multiple evidence records. The evidence record consists of a free-form, human readable statement and several structured elements. The statement consists of a few sentences written by a curator to summarize the clinical relevance of a variant according to evidence described in a particular publication. The curator attempts to concisely summarize the clinical assertion being made by the publication, as well as the nature of the evidence supporting that assertion and any caveats the reader should be aware of. The curator must also assign values for each structured element by evaluating details from the publication. These elements include evidence type, clinical significance, evidence direction, and others. Where possible, structured ontologies are used in the CIViC data model (e.g. the disease ontology for disease names). Dark blue boxes refer to primary CIViC entities and light blue boxes refer to external data.

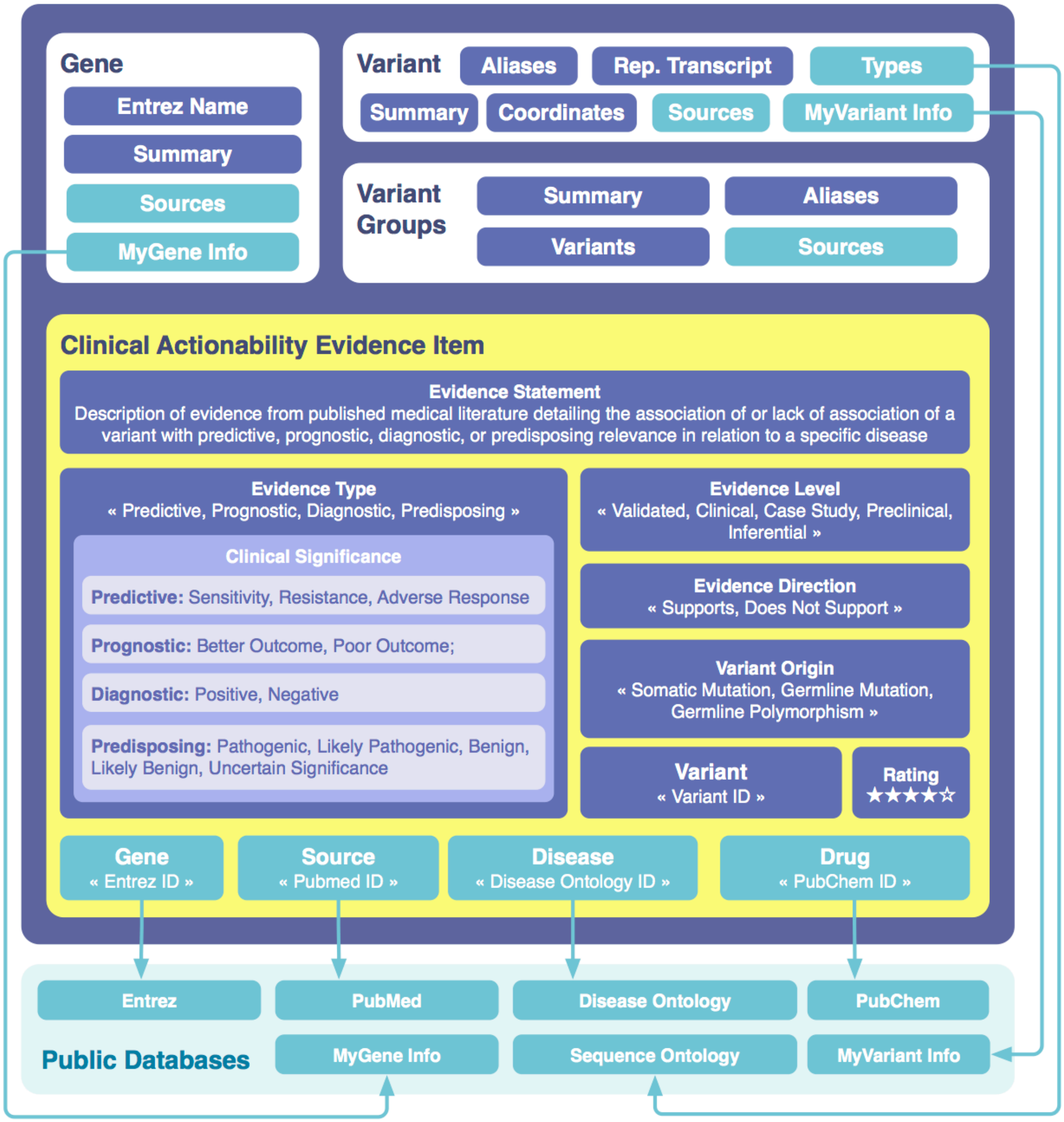

Supplementary Figure 3. Evidence level definitions and examples

Evidence levels defined in the CIViC data model are summarized below. Evidence levels are ordered A-E according to clinical utility (likelihood of relevance to a clinician reading a molecular report). A brief definition of each evidence level is provided along with an example obtained from www.civicdb.org. Updates to the CIViC data model (including to these evidence levels) will be maintained in the CIViC online documentation (https://civic.genome.wustl.edu/#/help/evidence). Additional examples of evidence records assigned to each evidence level can be obtained using the advanced search interface online: https://civic.genome.wustl.edu/#/search/evidence/.

Level	Definition	Examples and further comments
**A** Validated association	Proven/consensus association in human medicine.	*"AML with mutated NPM1" is a provisional entity in WHO classification of acute myeloid leukemia (AML). This mutation should be tested for in clinical trials and is recommended for testing in patients with cytogenetically normal AML.* Validated associations are often in routine clinical practice already or are the subject of major clinical trial efforts.
**B** Clinical evidence	Clinical trial or other primary patient data supports association.	*BRAF V600E is correlated with poor prognosis in papillary thyroid cancer in a study of 187 patients with PTC and other thyroid diseases.* The evidence should be supported by observations in multiple patients. Additional support from functional data is desirable but not required.
**C** Case study	Individual case reports from clinical journals.	*A single patient with FLT3 over-expression responded to the FLT3 inhibitor sunitinib.* The study may have involved a large number of patients, but the statement was supported by only a single patient. In some cases, observations from just a handful of patients (e.g. 2-3) or a single family may also be considered a case study/report.
**D** Preclinical evidence	*In vivo* or *in vitro* models support association.	*Experiments showed that AG1296 is effective in triggering apoptosis in cells with the FLT3 internal tandem duplication.* The study may have involved some patient data, but support for this statement was limited to in vivo or in vitro models (e.g. mouse studies, cell lines, molecular assays, etc.).
**E** Inferential association	Indirect evidence.	*CD33 and CD123 expression were significantly increased in patients with NPM1 mutation with FLT3-ITD, indicating these patients may respond to combined anti-CD33 and anti-CD123 therapy.* The assertion is at least one step removed from a direct association between a variant and clinical relevance.

Supplementary Figure 4. CIViC evidence classes and their relative potential to influence clinical actions and understanding of disease

The following diagram attempts to order each combination of evidence level (A-E) and evidence type (predictive, prognostic, diagnostic, or predisposing) according to their potential clinical relevance and actionability. 'Clinical relevance' refers to the contribution of the variant to clinical understanding of the disease and 'actionability' refers to the ability to identify a specific clinical action for a specific variant. In this assessment, validated predictive variants tend to be the most relevant and actionable, while inferential diagnostic are the least relevant. In general, higher evidence levels are more actionable and predictive assertions exceed prognostic and diagnostic evidence for clinical utility. While CIViC is designed to capture both supporting (positive) and refuting (negative) evidence, the following is an assessment of the likely utility of supporting evidence only.

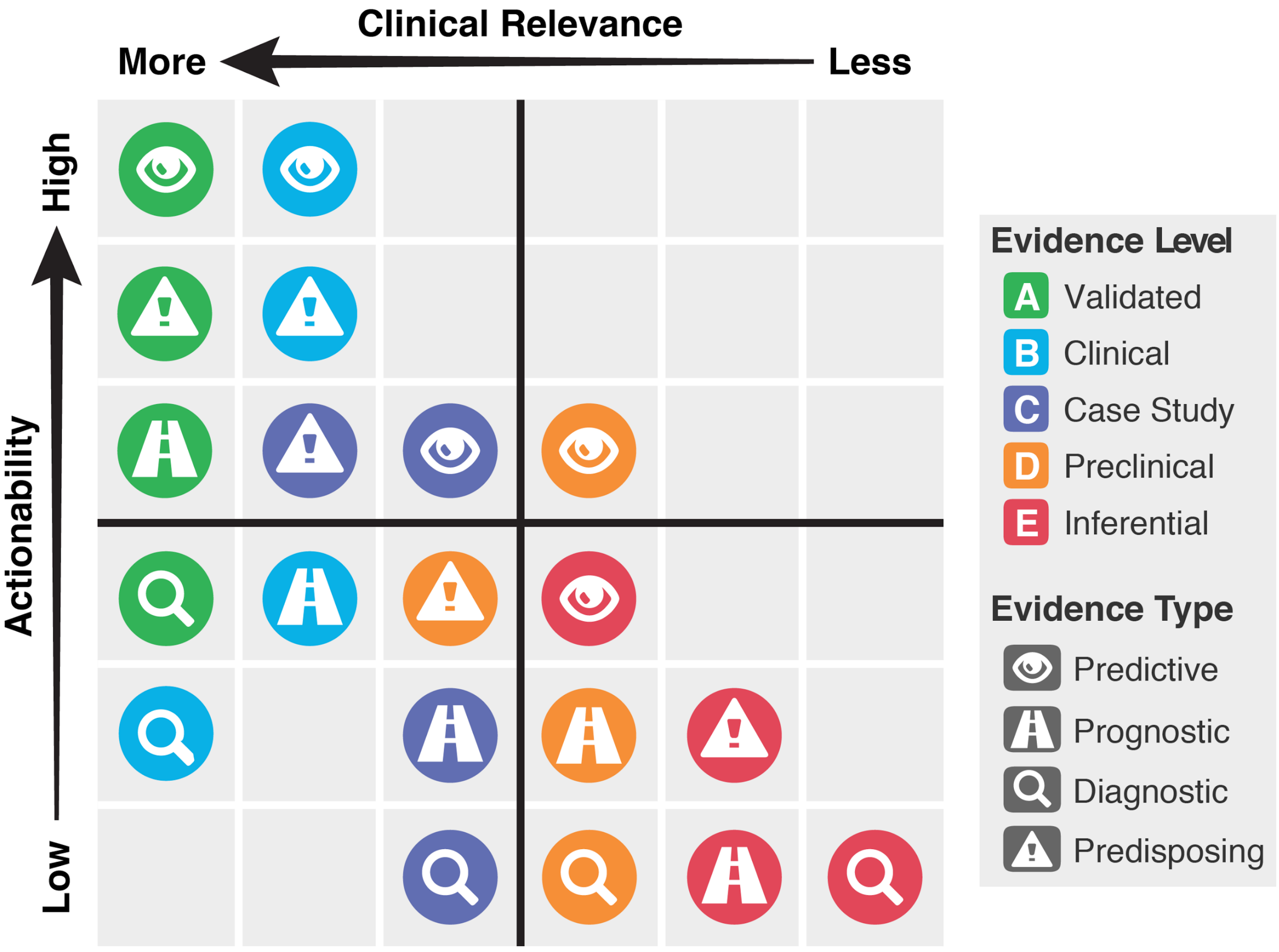

Supplementary Figure 5. CIViC database schema

A simplified schema representing the CIViC data model below provides all table names of the CIViC relational database (running on PostgreSQL). Polymorphic associations are used to relate core domain objects such as evidence records, genes, and variants to the tables that power on-site workflows like moderation and discussion. This allows for a significant reduction in the total number of tables required at the expense of database enforced foreign key constraints. In lieu of traditional foreign keys, validations in the application's business logic are used to enforce data integrity. Solid lines in the diagram indicate direct relationships in the database implemented by a local foreign key (for example, a variant has an evidence record identifier in the variants table, and thus a direct relationship). Dotted lines indicate relationships that exist indirectly (the relationship goes through an intermediate event with some conditions attached to it). For a complete schema including all fields and foreign key relationships, refer to the CIViC backend code repository: https://github.com/genome/civic-server.

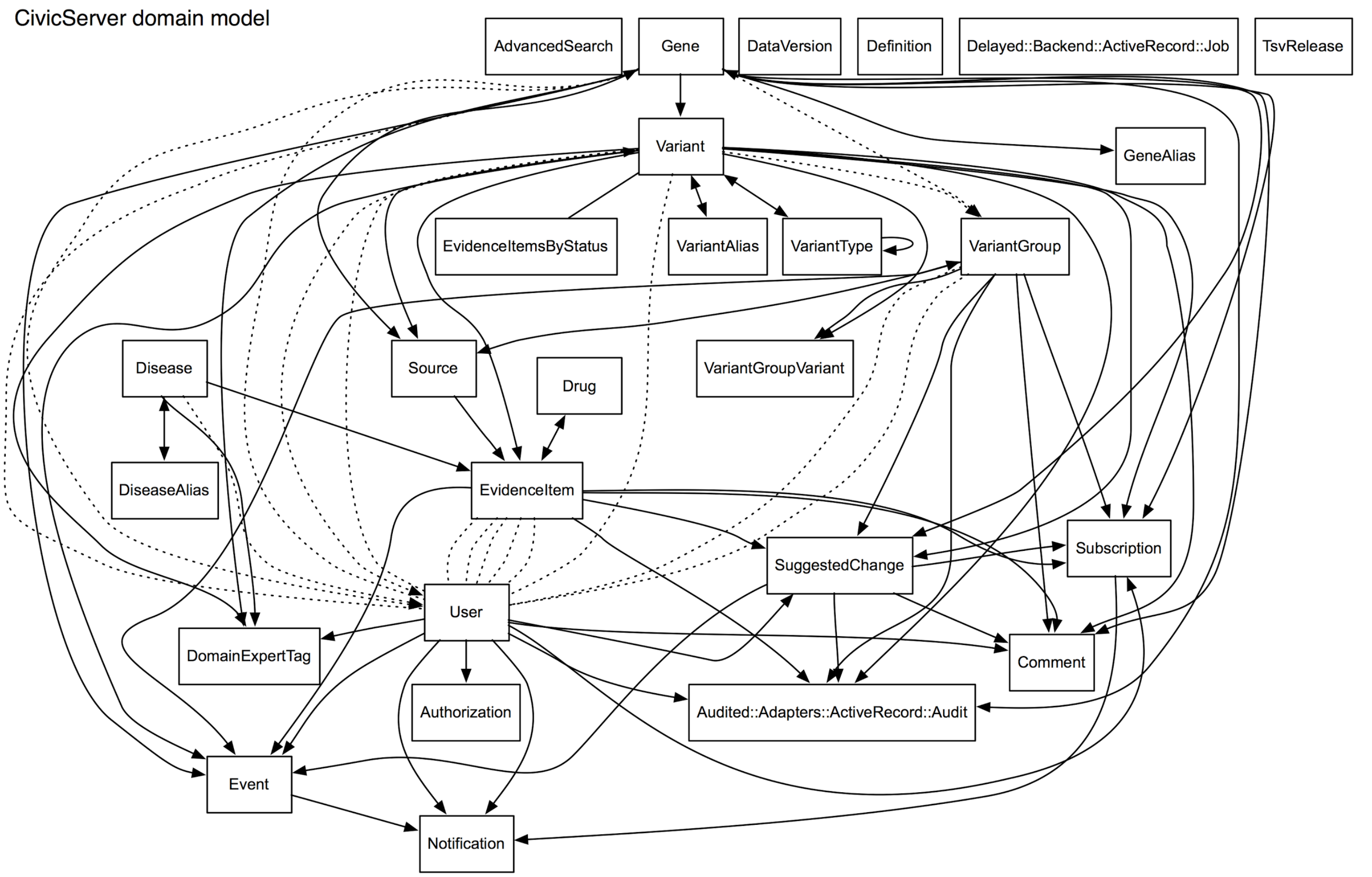

Supplementary Figure 6. Usage statistics and growth of content

A) CIViC content as of December 2016. B) Tracking of evidence statements within CIViC over time with respective contributions of internal (Washington University, ‘WashU’) and external (community) curation. C) Treemap with box size illustrating the relative number of visits (sessions) to the CIViC website www.civicdb.org from specific external organizations and colored by the average session duration (in seconds). Sessions from our own institute are excluded from this summary. D) Map illustrating the location where sessions originated. The size of the circles indicate the amount of traffic from each city. Dark blue indicates visits from a dense cluster of cities that are close to each other. To date, CIViC has achieved 39,881 visits from 16,484 unique visitors from 2,507 cities in 125 countries around the world.

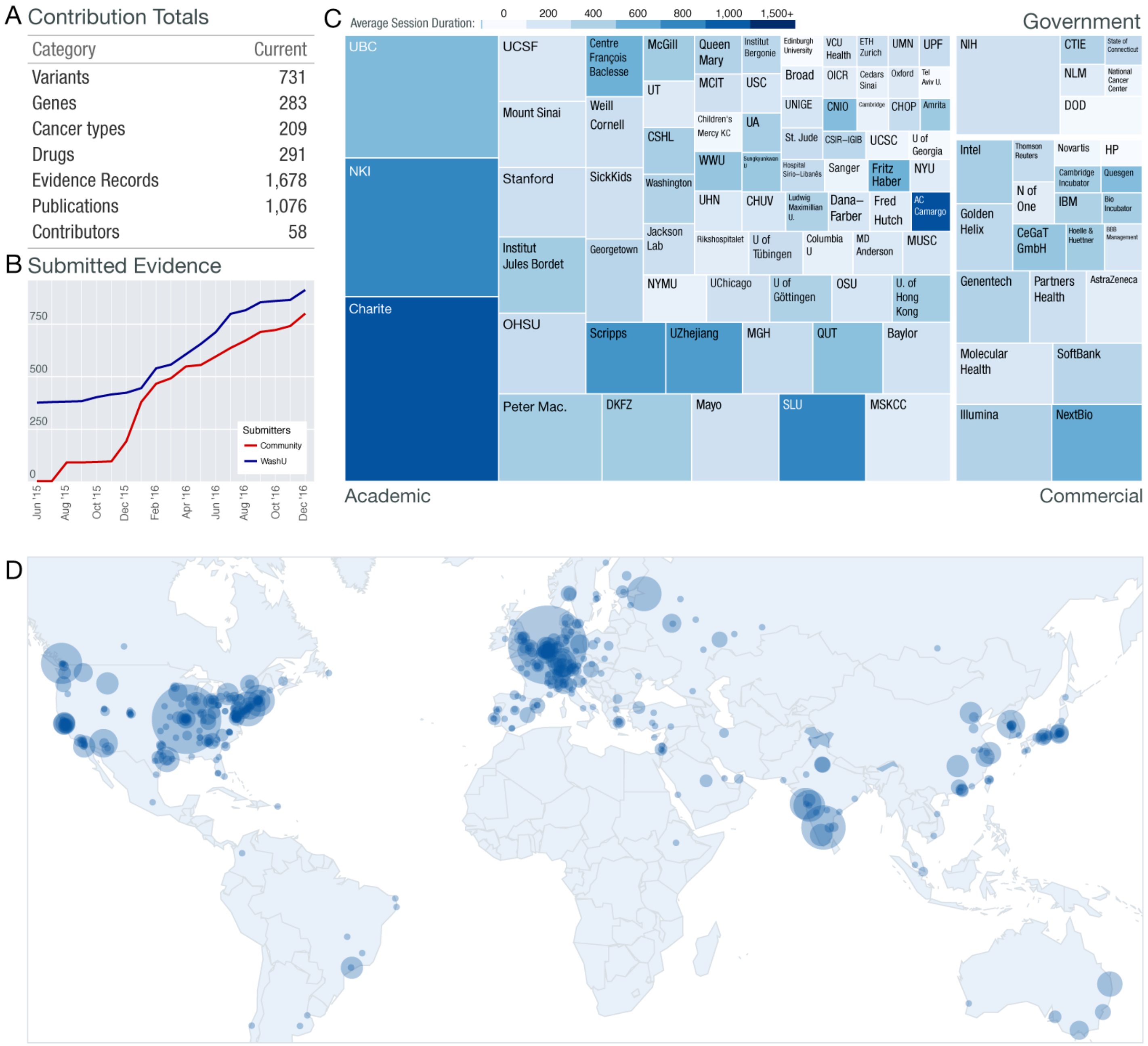

Supplementary Figure 7. Summary of current CIViC evidence records

The following panels briefly summarize CIViC evidence records at the time of publication. A) Total publications used in 1,703 evidence records, broken down by review status of the evidence record. Panels B-F further summarize these evidence records after excluding those that had a ‘rejected’ status (leaving 1,678 submitted or accepted evidence records). B) Evidence records broken down by evidence type and clinical significance. C) Evidence records broken down by evidence direction. D) Evidence records broken down by evidence trust rating. E) Evidence records broken down by evidence level. F) Evidence records broken down by variant origin.

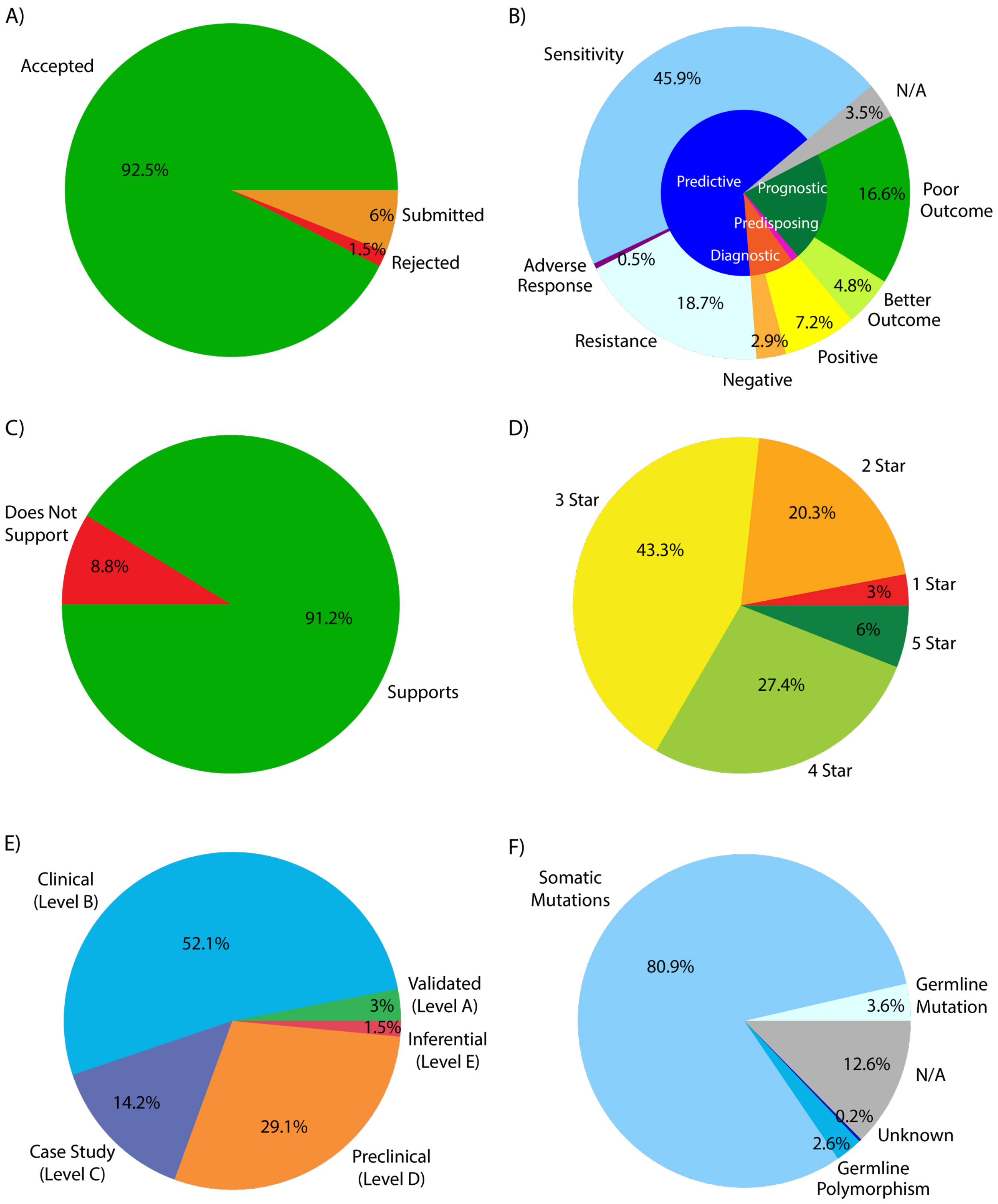

Supplementary Figure 8. Summary of the most curated drugs and diseases in CIViC
A summary of the drugs and diseases represented in CIViC evidence records ranked by the number of evidence records associated with each. A) The top 25 drugs were identified from 1,105 accepted or submitted evidence records of the predictive evidence type. The evidence records for these drugs are broken down by evidence level (left panel) and clinical significance (right panel). B) The top 25 cancer types (distinct disease ontology terms) were identified from all 1,678 accepted or submitted evidence records. The evidence records for these diseases are broken down by evidence level (left panel) and evidence type (right panel).

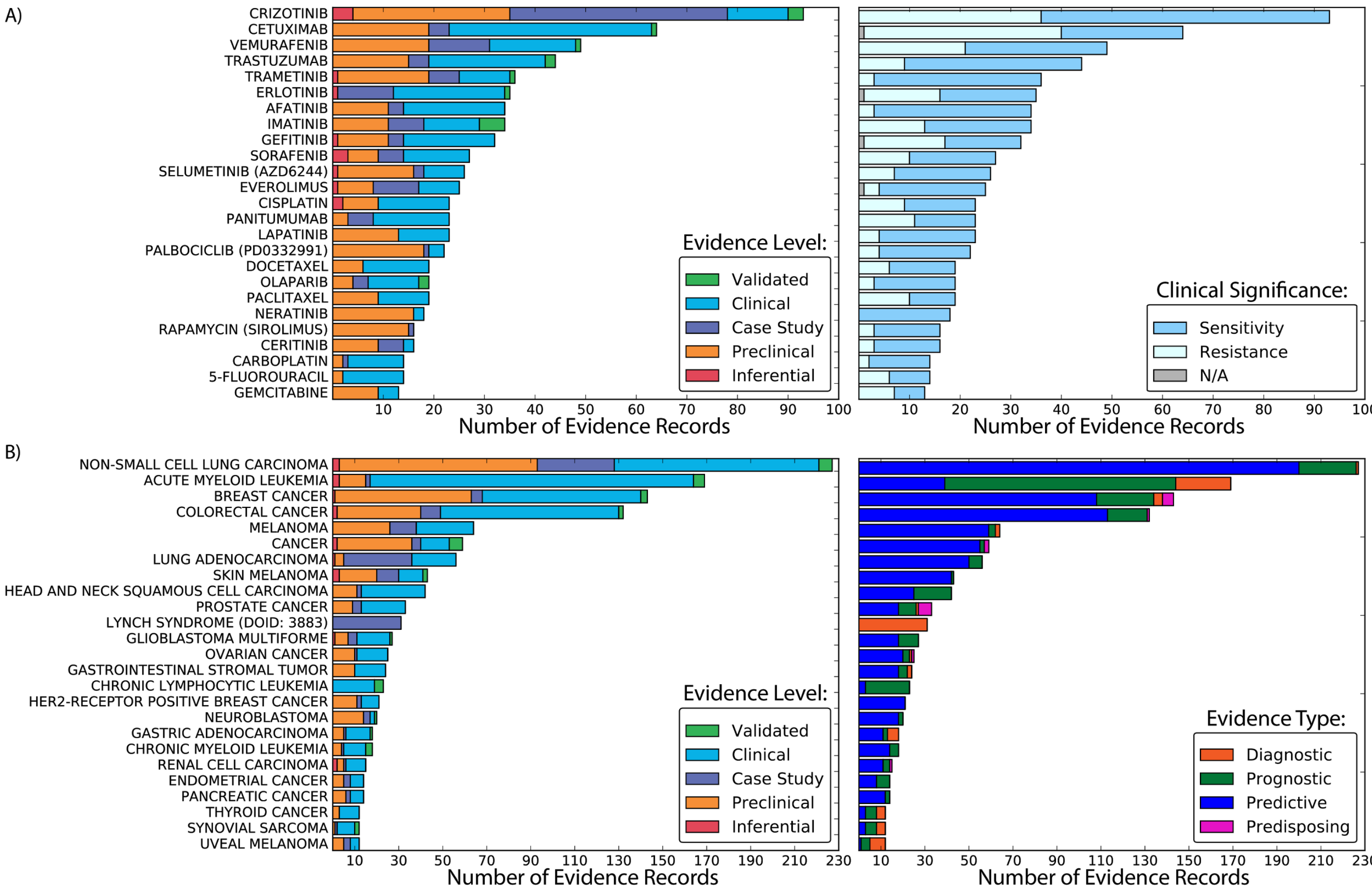

Supplementary Figure 9. CIViC evidence records summarized by literature sources

The published literature used to create all CIViC evidence records are summarized below. A total of 1,678 accepted or submitted evidence records were derived from 1,077 peer-reviewed publications. A) A histogram summarizing articles used in CIViC evidence records broken down by year of publication (and further divided according to their open versus closed access status). B) A histogram showing the distribution of number of evidence records obtained from single publications. Most publications yield only a single evidence record, but as many as 38 have been obtained from a single paper. C) Evidence records obtained from the top 25 journals most commonly mined in CIViC are summarized and broken down by evidence star rating on the left. The same evidence records are broken down by the evidence type on the right.

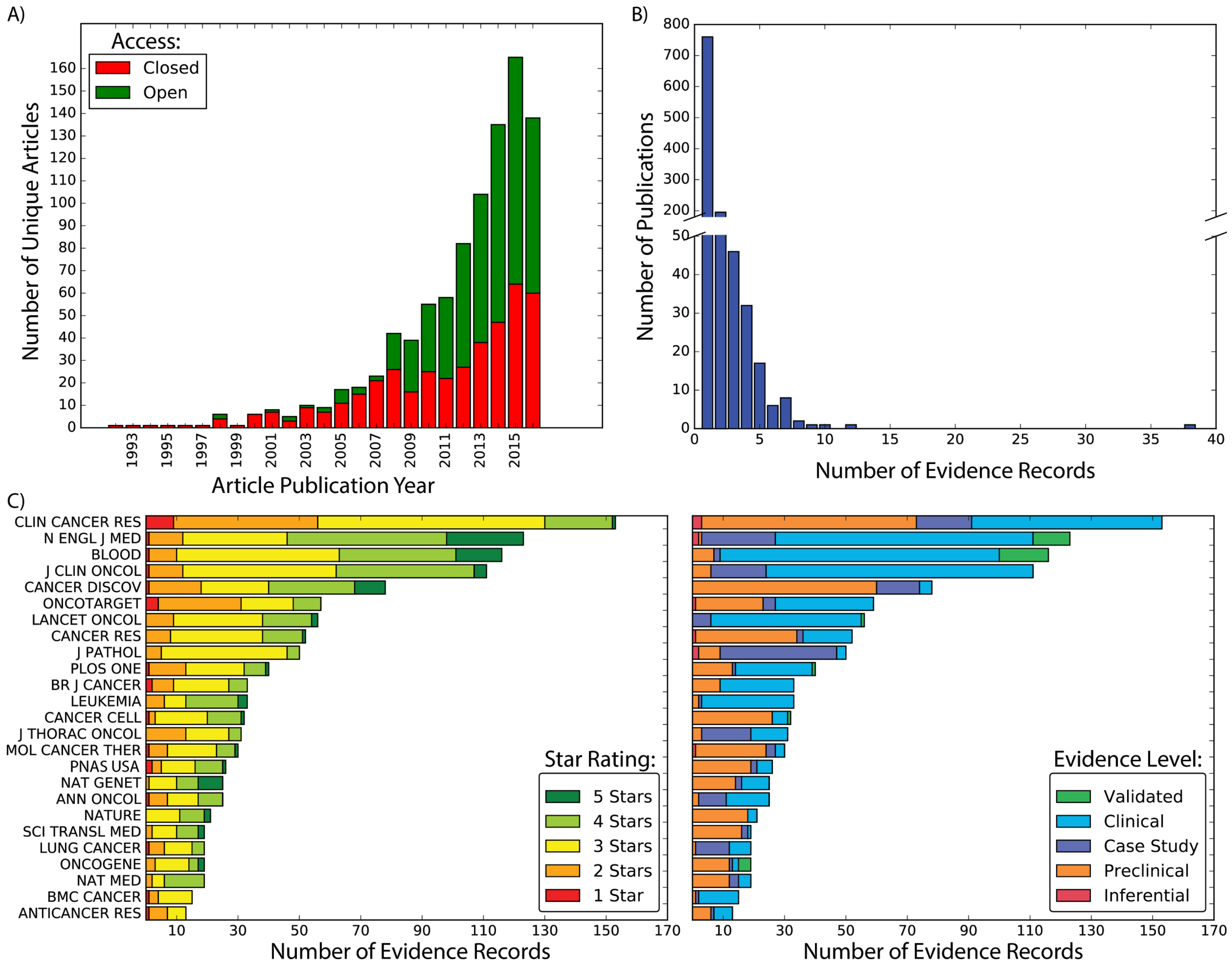

Supplementary Figure 10. The collaborative process and user roles in creating evidence
CIViC consists of an online web resource whose target audience is an international community of cancer researchers, clinicians, and patient advocates. Participants in CIViC fall into various categories with increasing privileges or capabilities in the interface. The first category and most basic level of user is that of ‘consumer’. Consumers may view, download and programmatically (via API) access all of the content of CIViC under the terms of the Creative Commons Public Domain Dedication license (CC0). No login is required to use CIViC. No requirement to login, fees, or other encumbrances will be introduced in future versions of CIViC. Consumers may not add, approve, edit, or discuss revisions of content in CIViC. The second category of users includes all those roles that do permit modification and discussion in the site: ‘curators’, ‘editors’, and ‘administrators’. ‘Curators’ may add new evidence records describing clinical relevance of variants, add or improve variant/gene summaries, and discuss existing content. While comments/discussion are automatically accepted, additions and revisions to existing content are initially entered in a pending state and must be approved prior to acceptance in CIViC. Rejected content is not deleted and may be revived after further discussion and revision. Editors have the additional capability to approve or reject additions and revisions of content. However, an editor cannot approve their own submissions or revisions, meaning that all content in CIViC must be created in collaboration between at least two members of the community. Editors are selected by a committee of existing editors, based on direct knowledge of the editor’s expertise or by promotion from curator after demonstrating extensive high quality contributions to CIViC. Finally, administrators have the abilities of editors but may also change user roles and use advanced site management utilities (e.g. merging duplicate records).

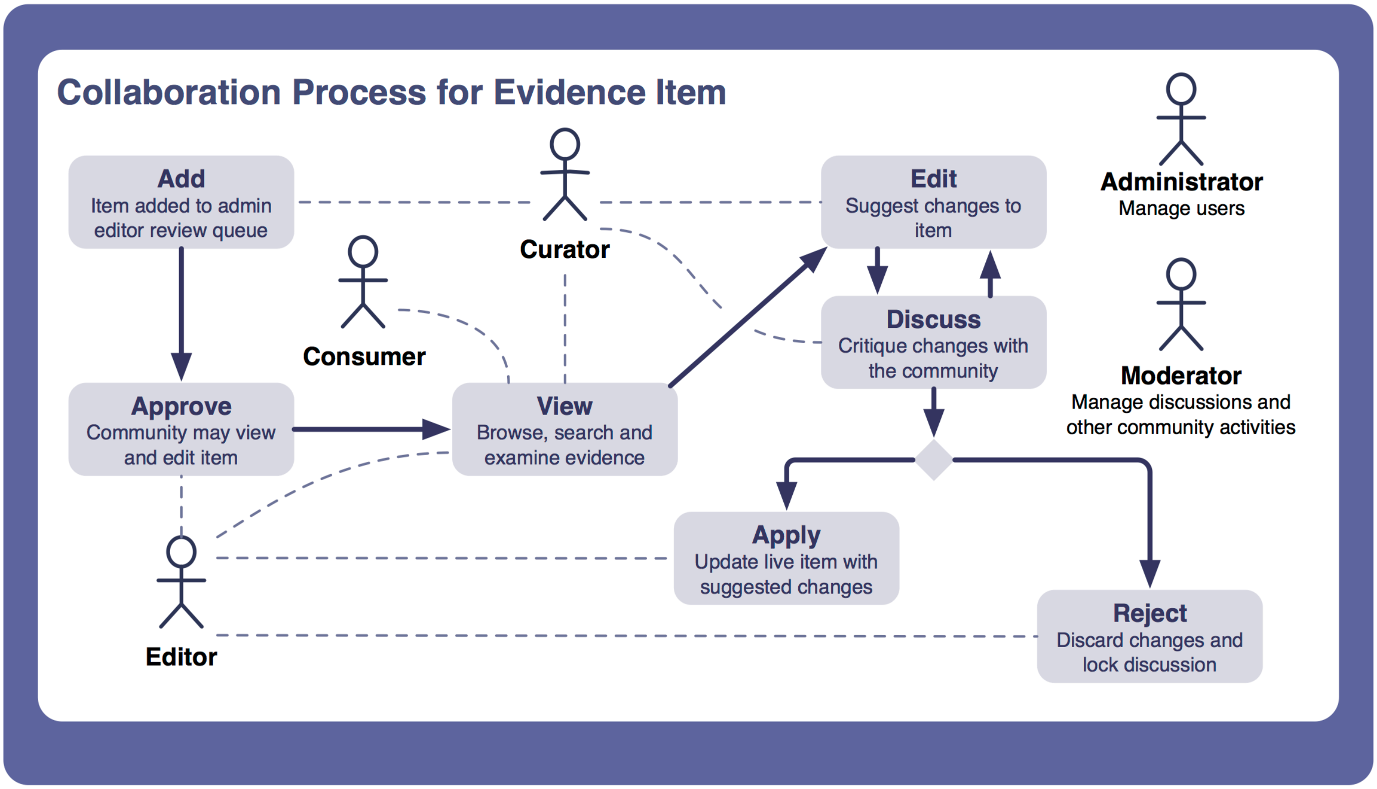

Supplementary Figure 11. Screenshot of the editor view for a submitted evidence record
Every new evidence record and any revision of existing content in CIViC must be approved by at least one independent editor prior to acceptance. The following screenshot shows a new evidence record submitted by a curator that is awaiting review by an editor. The following URL will display the live version of this example: https://civic.genome.wustl.edu/links/variants/34

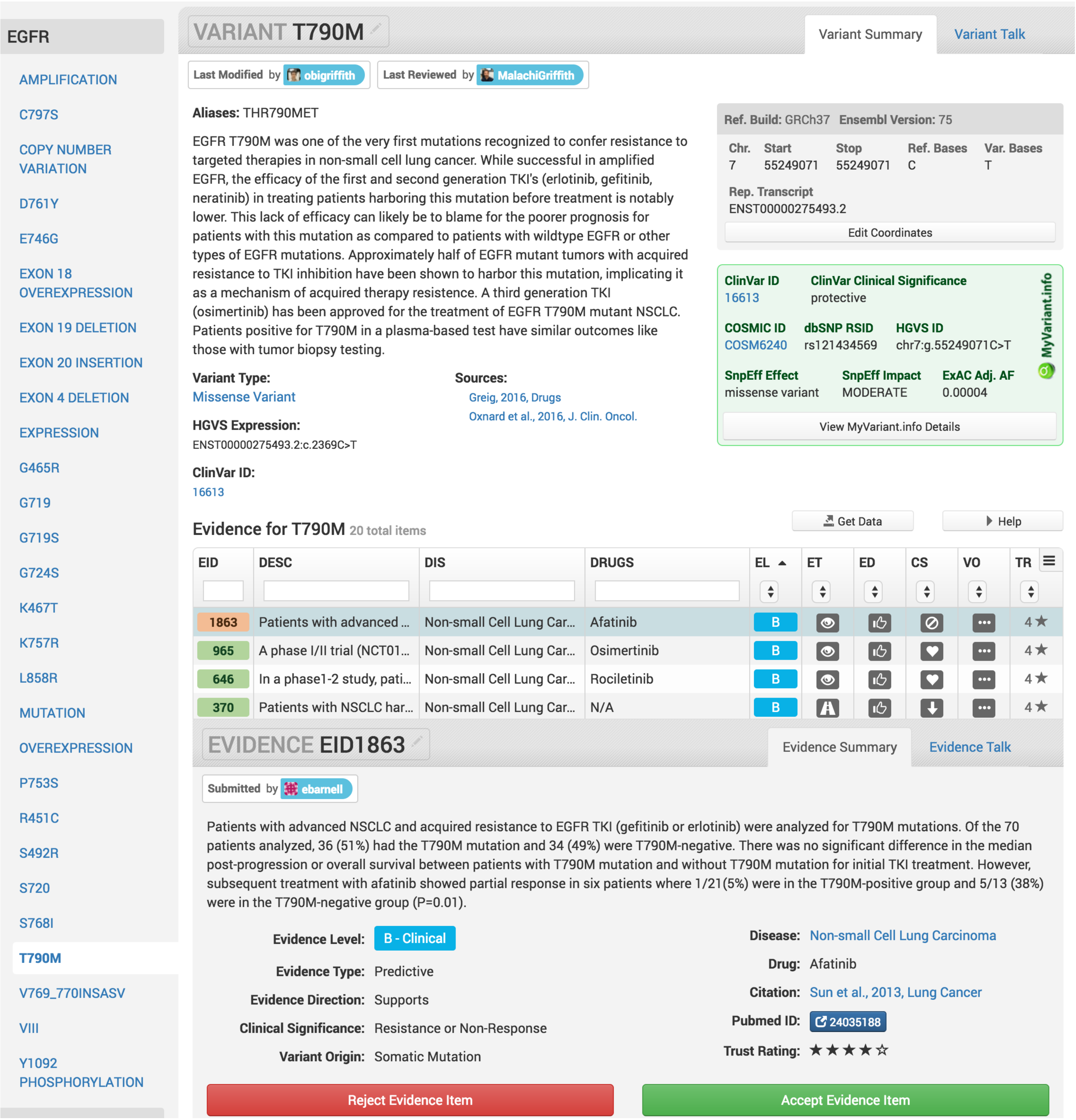

Supplementary Figure 12. Screenshot of the editor view for a pending revision
After proposing a revision to existing content, a contributor is presented with a summary of the fields they are proposing to modify. An independent editor must approve these revisions before they are displayed in the canonical CIViC results (the web interface and API).

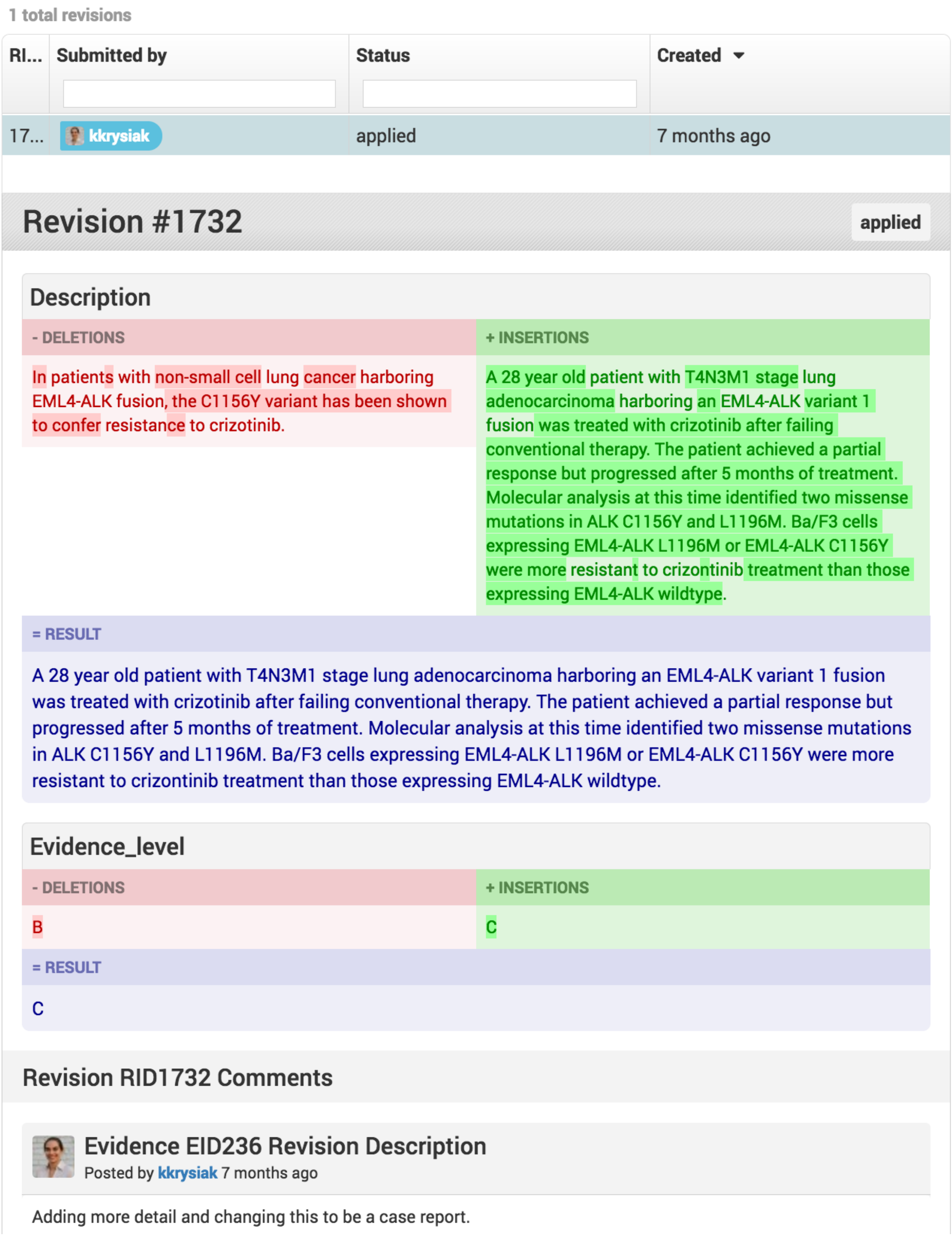

Supplementary Figure 13. Screenshot of a complex evidence query

CIViC has an advanced search interface that currently supports complex queries for evidence records and variants. An arbitrary number of query conditions can be set and the query can be configured to match any one, or all of these conditions. Evidence records can be queried by sixteen variables including disease, variant name, publication ID, evidence type, evidence level, trust rating, curator name, etc. In the following screenshot, the advanced search interface is being used to retrieve all evidence records that correspond to variants involving the gene *ALK,* where the evidence type is 'Predictive', and the drug involved is alectenib. From this query, 13 evidence records are returned and sorted according to their quality level (evidence level, and trust rating). The standard CIViC evidence datagrid is used to display a summary of the 13 evidence records including: evidence identifier (EID), gene name, variant name, evidence statement (DESC), cancer type (DIS), drugs, evidence level (EL), evidence type (ET), evidence direction (ED), clinical significance (CS), variant origin (VO), and evidence trust rating (TR). The 'Help' button provides a comprehensive legend of all abbreviations, symbols, and colors used to encode information in the evidence record summary. Clicking any row will take the user to the comprehensive display for that evidence record. Every advanced search generates a unique URL that can be used generate an updated result or easily share the result with a colleague. For example: https://civic.genome.wustl.edu/#/search/evidence/fbf0df08-0211-4e55-b4e7-d103d76d0b59.

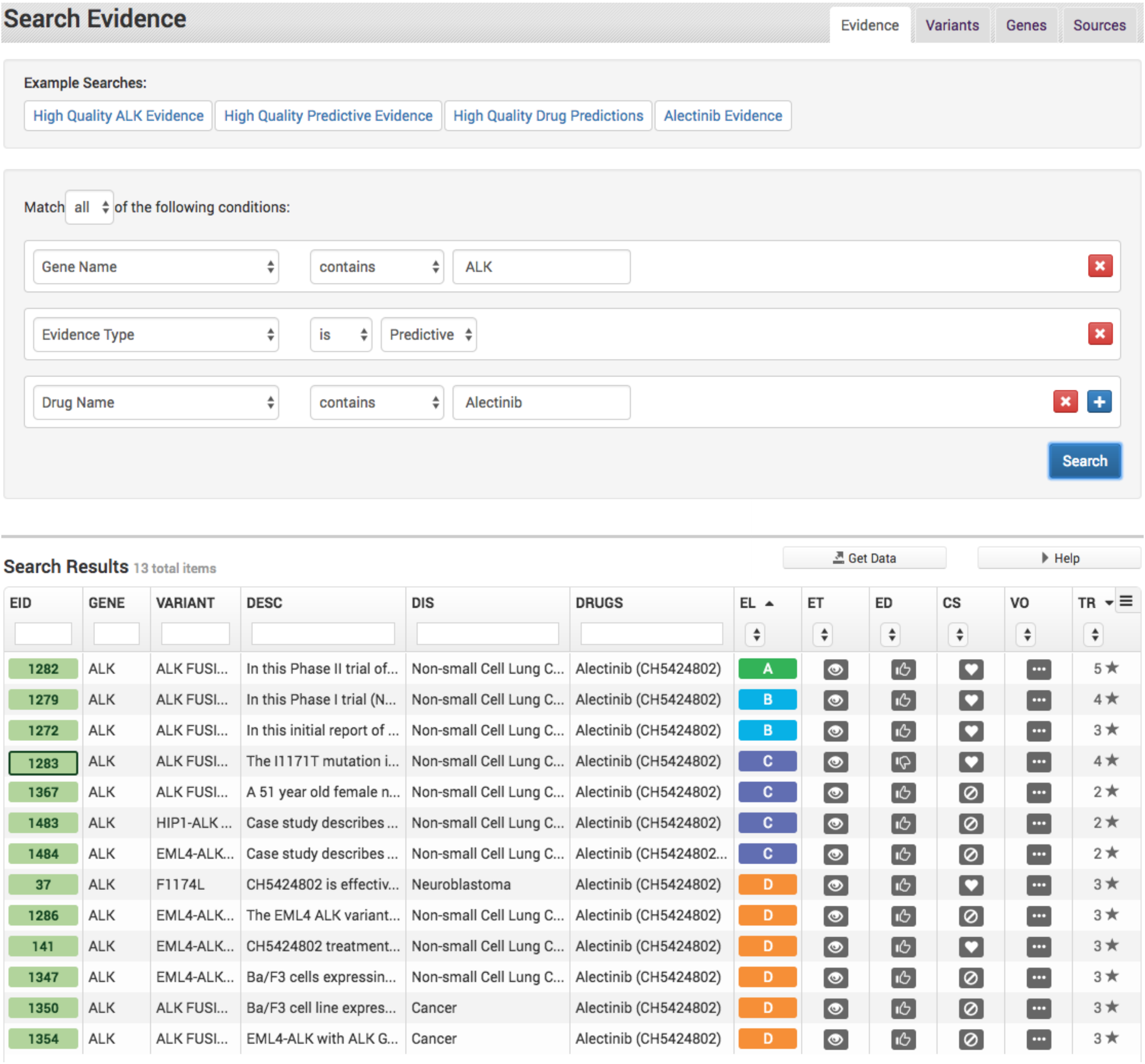

Supplementary Figure 14. Screenshot of the source suggestion queue
CIViC includes a “source suggestion queue”. This feature allows CIViC external domain experts to quickly and easily add important publications (using PubMed ID) to a queue for later generation of evidence records by the curation team. In addition to PubMed ID, an entry to the queue contains a free text field where submitters can add comments to help guide curation efforts related to each publication. Optional fields available when creating an entry for the publication queue are gene, variant, and disease. Action buttons allow curators to add new evidence records for each publication suggested (yellow), reject the suggestion (red), mark the suggestion as completed (green), or re-activate the source in the suggestion queue (grey).

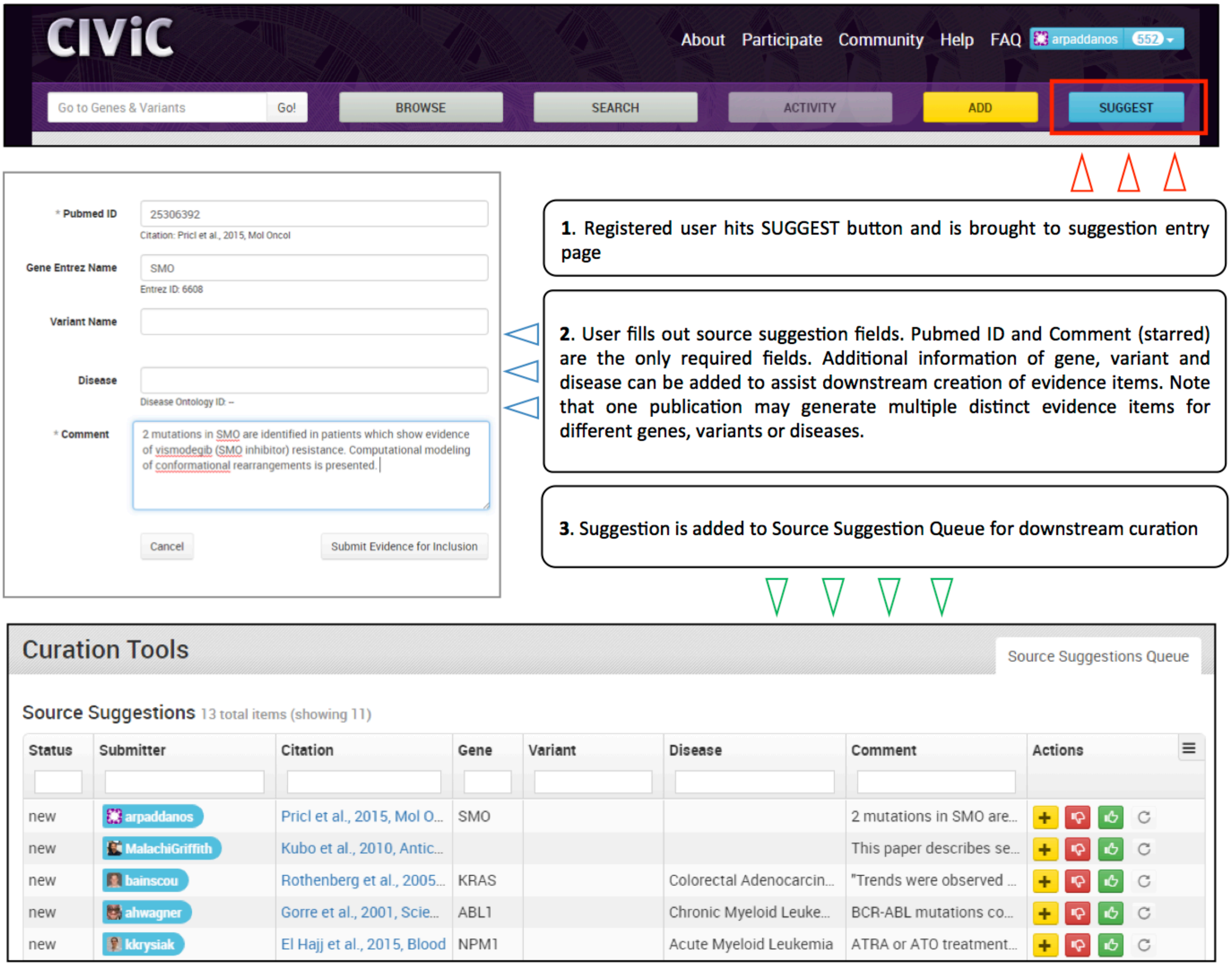

Supplementary Tables

Supplementary Table 1. Related resources

This table compares CIViC to other resources with regard to their curation model, ability to view content without registering, existence of a public API, ability to download bulk data, open licensing of the code and content, and various technical features.

This table can be downloaded as a spreadsheet from the journal's website.

Alternatively, a live version that will be updated as these resources develop can be found here: https://goo.gl/5WAZmd

Supplementary Table 2. Literature covered by CIViC compared to related resources

This analysis was performed using data obtained from CIViC and seven related resources in July 2016. At that time, CIViC contained curated evidence records obtained from 895 peer-reviewed publications. A summary of the overlap between these publications and those curated by each of the related resources is provided below. Refer to **Supplementary Table 1** for extensive details of each resource.

This table can be downloaded as a spreadsheet from the journal's website.

References

1. Wagner, A.H. *et al.* DGIdb 2.0: mining clinically relevant drug-gene interactions. *Nucleic Acids Res* **44**, D1036-44 (2016).
2. Ritter, D.I. *et al.* Somatic cancer variant curation and harmonization through consensus minimum variant level data. *Genome Med* **8**, 117 (2016).
3. Xin, J. *et al.* High-performance web services for querying gene and variant annotation. *Genome Biol* **17**, 91 (2016).
